# Design and implementation of a web-based patient registration system in a single-centered tertiary care hospital of coastal Karnataka

**DOI:** 10.1186/s12913-026-14299-3

**Published:** 2026-03-10

**Authors:** Ashwitha N. Amin, G. Somu, Prajwal L. Salins, Roshan David Jathanna

**Affiliations:** 1 Prasanna School of Public Health, Manipal Academy of Higher Education, Manipal, India; 2 Department of Hospital Administration, Kasturba Medical College, Manipal Academy of Higher Education, Manipal, India; 3 Department of Health Information Management, Manipal College of Health Professions, Manipal Academy of Higher Education, Manipal, India; 4 Manipal Institute of Technology, Manipal Academy of Higher Education, Manipal, India

**Keywords:** Outpatient registration, Process optimization, Time and motion study, Hospital information systems (HIS), Patient satisfaction, mHealth

## Abstract

**Background:**

Prolonged waiting times for outpatient registration remain one of the significant challenges in tertiary care hospitals, leading to patient dissatisfaction and operational inefficiencies. The use of traditional paper-based registration systems is one of the reasons for the delays, especially during peak hours. The aim of this study was to create a web-based outpatient registration system in order to make waiting time shorter and to improve service efficiency.

**Methods:**

This prospective observational study was conducted in two phases at Kasturba Hospital, Udupi. Time and motion study was done on 50 patients in Phase I, and service time for 20 patients was measured using stopwatch-based observation. A structured, validated questionnaire was administered to 200 patients to capture satisfaction levels, awareness, and willingness to use a digital registration system. During Phase II, an online registration platform was developed using the waterfall model, which included planning, system design, programming, testing, and maintenance. The Django framework was used to develop the system with MySQL as the backend database. To ensure the smooth integration with the existing hospital system, a special browser plugin was created.

**Results:**

The time it took to register patients averaged 17 min and 25 s in total, and it took 6 min and 2 s on average for the staff to serve the patients. Among the 200 patients surveyed, the current system was satisfactory for 74.5% of them, while 25.5% voiced their complaints mainly because of the long waiting lines. The majority, that is, 58% of patients were unaware of the online registration facility, however, 90.5% of them were ready to utilize such a system if it was introduced. Moreover, 83% of the respondents thought that the online registration system would allow them to spend less time waiting. A digital solution was created and integrated into the hospital’s system for pilot testing.

**Conclusion:**

This study reveals that there is both the need for and readiness to undergo a digital transformation of the outpatient registration process. The system for online registrations developed as part of this study is both technically viable and appreciated by the patients. Wider implementation and awareness-building, which help to decrease waiting times and boost operational efficiency, lead to greater patient satisfaction in tertiary care facilities.

**Supplementary Information:**

The online version contains supplementary material available at 10.1186/s12913-026-14299-3.

## Introduction

Hospitals play a pivotal role in delivering comprehensive healthcare services through inpatient, outpatient, and emergency care [1]. The Outpatient Department (OPD) is among the places where most of the patients visit the hospital for the first time and is often considered the hospital’s, shop window [2]. The OPD reflects the overall quality, efficiency, and responsiveness of the entire healthcare facility, thereby influencing public perception and patient satisfaction. Literature shows that the quality of the OPD services has a considerable impact on the overall assessment of the hospital’s healthcare delivery standards [3, 4]. Traditionally, medical professionals assessed quality based on their standards of practice. Over time, the assessment methods have changed dramatically and have come close to the patient-centered care approach where the shift turned from quality indicators to user experience and satisfaction with quality as its prime indicators [5]. Thus, it is necessary to conduct enhancement programs in outpatient services to provide timely, accessible, and efficient care.

One of the most consistent challenges encountered in OPD is the long, time taking and tedious registration process. With patients having to fill the registration forms manually by themself and then standing in very long queues before even being treated, which indeed increases the waiting time for treatment, resulting in operational inefficiencies due to patient dissatisfaction [6, 7]. In large tertiary care hospitals, especially in resource-deficient areas like coastal Karnataka, such inefficiencies occur due to higher patient volumes and fewer manpower administration. With digital technology making its way in every sector, its potential for application in changing hospital operations is gaining appreciation. The web registration system that is accessible through mobile devices has the capability to facilitate the processes of patient entry and decrease contact between persons thus cutting down on clerical errors and improving the efficiency of workflow [8].

**Fig. 1:**
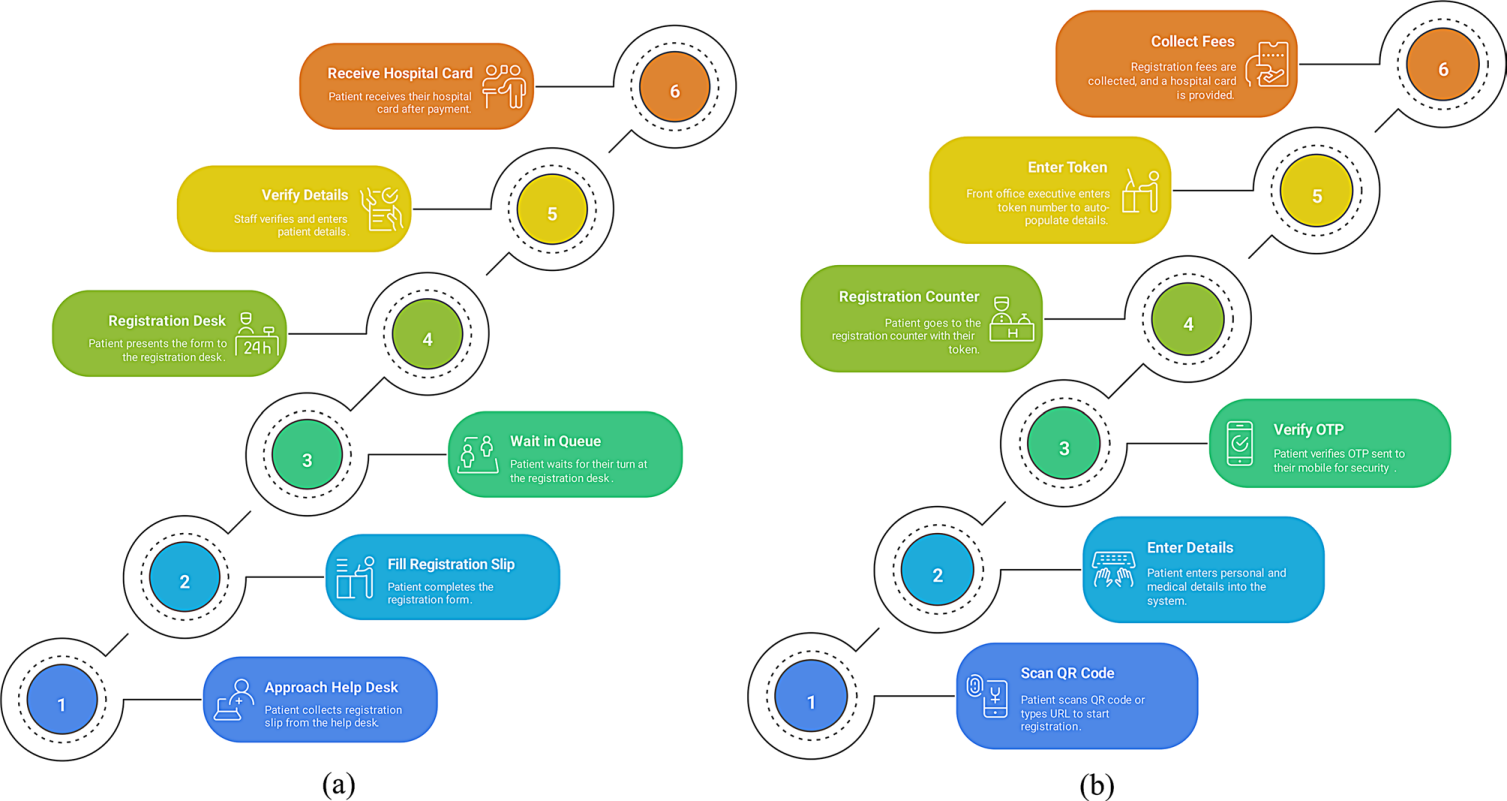
Workflow of (**a**) Paper based and (**b**) Online registration system overview

The transition from conventional outpatient registration to mobile-based registration systems serves as a vital advancement which enhances overall efficiency within the healthcare delivery system. The traditional workflow consisted primarily of manual data entry, queuing, and issuing physical tokens [9, 10]. In contrast, the mobile system enables remote data entry, token auto-generation, and direct access to outpatient services. Digitization aims at streamlining patient movement, freeing up registration areas, and providing quicker service. The highlights are that with fewer checkpoints and delays, the mobile system will optimally contribute towards state-of-the-art patient-centered care and infection control guidelines, now much more relevant in the post-pandemic healthcare setup (Fig. 1). The objective of this study focuses on the development and implementation of a web-based registration platform for the Outpatient Department of the tertiary care hospital in coastal Karnataka. The general objective of this study is to design a digital solution that would allow patients to register for outpatient services from their smartphones. The study aimed to assess the current average waiting time at the registration counter, evaluate patients’ willingness to adapt to the proposed registration system, and determine the intervention’s impact on the overall effectiveness of the registration process. Waiting time was assessed through direct observation using stopwatch-based time recordings. At the same time, patient feedback was collected to assess user acceptance and support decision-making on the suitability of the proposed solution from an operational perspective.

Reducing outpatient waiting time are the focal point of research discourses in health systems, with waiting times regarded as commonly perceived key performance indicators in relation to hospital-services quality [11–13]. By observing and recording time spent during various stages of the registration process, this study aims to provide a baseline assessment and demonstrate the tangible benefits of a digital alternative. Beyond convenience, the web-based registration system also supports infection control efforts, particularly in light of the COVID-19 pandemic, by reducing crowding in hospital waiting areas, historically known clusters of transmission [7]. The integration of digital health tools into hospital infrastructure is no longer a luxury but a necessity, and this initiative serves as a scalable model for similar healthcare institutions aiming to modernize their outpatient services [14].

## Methodology

This prospective observational study was conducted at Kasturba Hospital, tertiary care teaching hospital in a semi-urban area setting, located in Udupi District, Karnataka, India. The study was designed to be conducted in two distinct phases. To assess both the existing outpatient registration process and also to develop a new and improved digital alternative in the form of a mobile-based online registration system. The study site was basically opted based on its daily high outpatient volume, its accessibility to research team and its representativeness of public healthcare infrastructure in the coastal region. These factors made it an apt setting to evaluate the feasibility of a digital intervention and to assess operational inefficiencies in outpatient registration. Patients under 18 years old were excluded because they are legally minors and could not participate in the study for ethical reasons. Patients aged 65 and older were excluded to ensure uniformity in digital literacy levels and cognitive abilities relevant to evaluating registration workflows and digital service interactions. Patients aged 65 or older often require caregiver assistance or follow different registration pathways, which could introduce variability unrelated to the system being assessed.

Sample size: This research was conducted using a cross-sectional observational approach, and the sample size for the patient satisfaction survey was determined based on the expected proportion of patients who are dissatisfied with the outpatient registration process in place. The required sample size was calculated using the standard formula for proportions given an expected satisfaction rate of 70% (based on preliminary observations), a 95% confidence level, and a 7% margin of error. For non-responses or incomplete entries, the final target sample size was set at 200 respondents. This number was considered enough to investigate the patient satisfaction patterns and the disposition to accept digital solutions. The time-motion study involved 50 patient observations and 20 staff service time recordings which was used as observational data according to sample sizes defined in earlier studies of hospital inpatient workflow analysis [10, 15].

Statistical Method used: Quantitative data collected from the time-motion study and patient satisfaction survey were entered and analysed using Microsoft Excel (Microsoft Corp., Redmond, WA, USA). Descriptive statistics, including means, percentages, and frequencies, were used to summarize the average time spent during registration and the distribution of patient responses across various survey categories.

### Phase I: Time and motion study and patient satisfaction assessment

During the first phase there was a time and motion study to find out the duration of each activity in outpatient registration processes. A total of 50 patients were selected as a convenient sample for observation and recording of the time they spent at the registration counter. Observations were done with a stopwatch, and each patient was timed from when the person got to the counter till the completion of registration proceedings. During the same time, an additional 20 patients were being observed to determine the average time staff took to provide service at the registration counters. This observation helped identify time-consuming operations and service bottlenecks. After their observation analysis, a validated structured questionnaire was given to 200 patients for evaluating their satisfaction levels regarding the time spent in the Outpatient Department (see Supplementary file 1). The patient satisfaction questionnaire was developed using literature examination and expert advice assessment methods that fit with hospital registration and service delivery standards. A group of ten experts which included four health information professionals, three hospital operations specialists, and three hospital administrators evaluated the content validity. The researchers used content validity index and content validity ratio and modified kappa statistics to assess item relevance and item clarity. The item-level CVI (I-CVI) ranged from 0.90 to 1.00 at the same time the scale-level CVI (S-CVI/Ave) achieved 0.97 which demonstrated excellent content validity. CVR values ranged from 0.80 to 1.00 which exceeded Lawshe’s recommended threshold of 0.62 for ten experts. The modified kappa values demonstrated excellent inter-rater agreement because they ranged from 0.90 to 0.99. The patient satisfaction questionnaire was conducted during the period after the electronic registration system had been introduced in order to evaluate how users experienced the system and how satisfied they were with the digital registration process and its perceived efficiency. The questionnaire included questions that assessed the satisfaction level of applicants throughout the registration process while they succeeded in navigating system operations and understanding process details. The researchers collected responses in an anonymous manner to protect participant confidentiality before using the data for analysis during the second stage of the intervention.

### Phase II: Development of the web-based online registration system

The second phase of the research required development of an online registration system through Waterfall Software Development Life Cycle (SDLC) methodology. The Waterfall methodology functions as a sequential development process which consists of six distinct steps that include planning system design programming testing installation and support.

Planning: The main goal during the planning stage was to apprehend the current manual registration procedure and determine the difficulties encountered by both patients and management. The project required multiple analyses which examined problems together with data software and hardware requirements. The Software Requirements Specification (SRS) document served as the output for this stage. The document specified all system functions and performance requirements through a standard language which helped technical developers and hospital stakeholders understand each other.

**Fig. 2:**
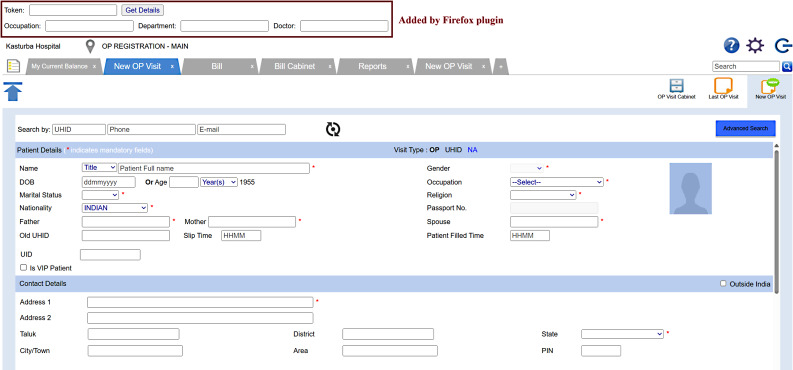
Hospital information system page with Firefox plugin

System Design: The system design phase used collected requirements to develop both a high-level system architecture and a detailed low-level design. This process required the development of system flow descriptions together with user interface design and database structure definition and module interconnection mapping. The intent was to supply a logical framework that would carry through the implementation stage with least uncertainty. The results of this stage were documented in a Software Design Document (SDD), which became a complete reference for developers, encompassing both architectural decisions and specific functional pathways. Data needed were patient name, age, marital status, occupation, parent details, address, contact number, the department for consultation which are needed for the registration process. The system is a responsive web application developed using the Django framework, with Python as the programming language and MySQL as the backend database [16]. It enables patients to register through a user-friendly interface accessible across devices. For integration at the registration counter, a Firefox browser plugin was developed using JavaScript (Fig. 2).

**Fig. 3:**
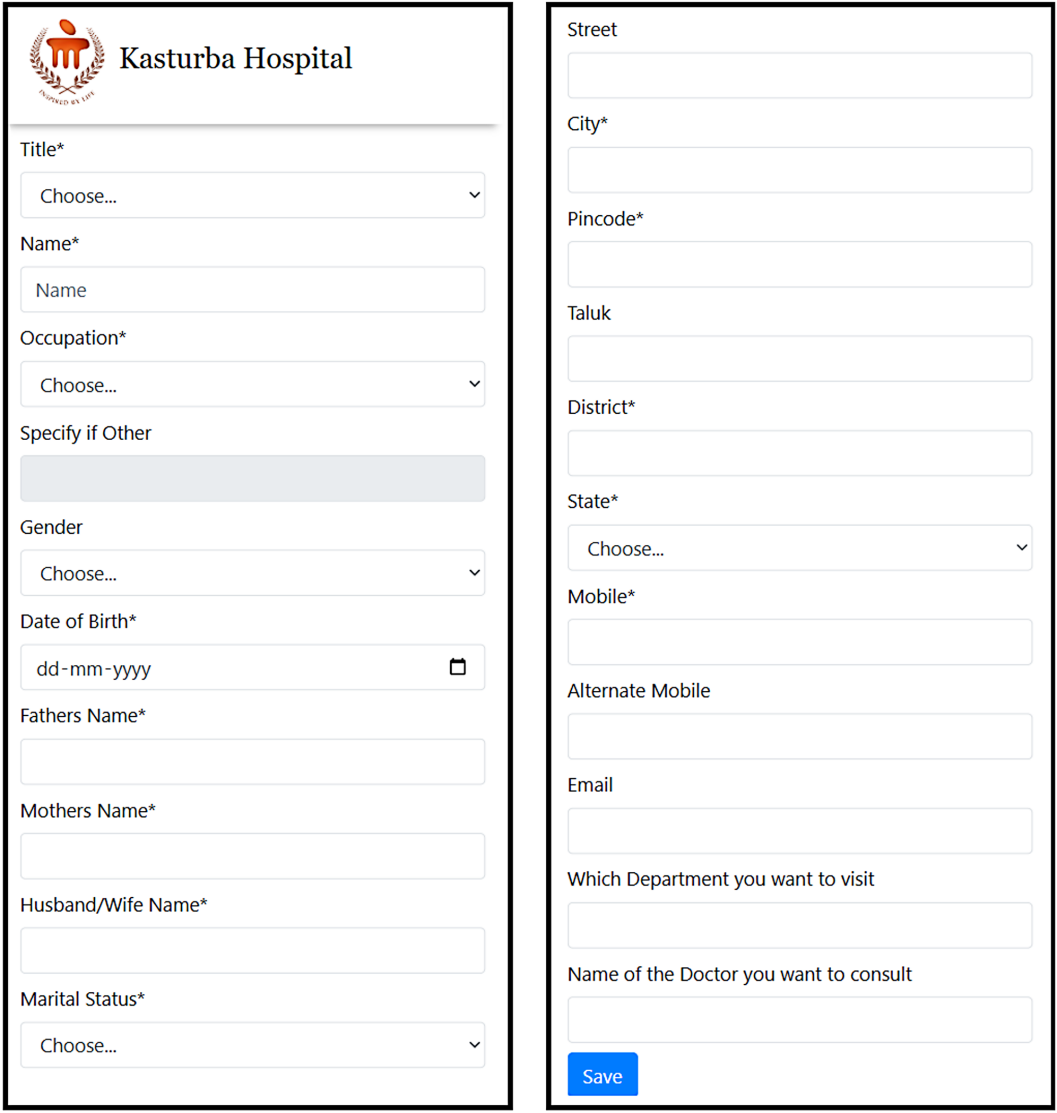
Patient registration form

Programming: The implementation stage consisted of the development of a functional web-based patient registration platform, which was done by converting the SDD specifications. Having comprehensive design documentation made the programming process run smoothly and so the requirements were met from the beginning and development errors were reduced to a minimum. The platform resulting from this development grants patients the opportunity to submit their registration info over the internet via a web interface that adapts to different screen sizes. The patient registration form is shown in Fig. 3. An automatic generation of a unique token number occurs after the submission of the form. This token can then be shown at the hospital’s registration desk. The registration personnel enter the token number into a custom-built Firefox browser plugin, which securely retrieves the submitted information and automatically populates the required fields in the hospital’s existing registration system. This approach eliminates the need for manual data input while it decreases mistakes that occur during transcription and it delivers faster service to customers. The system retains complete records of token usage and data changes and timestamp information in addition to the registration process for purposes of accountability and traceability and quality control. The system enables safe transfer of collected information which organizations can use for their institutional reporting needs and their operational planning activities. The system was created to work with the hospital’s existing operational processes because it does not need major system upgrades and use existing software.

Testing: After the development was done, the system went through a series of tests to check the functionality, reliability, and compatibility with various devices and browsers. The unit test verified the functional parts, and the integration test confirmed the proper interaction of modules. User acceptance testing (UAT) was performed with hospital staff and a group of patients to check the system’s friendliness and efficiency in the real world. No issues left unattended before the release.

Maintenance: The system operations from deployment to security and performance and system capacity require ongoing security activities which occur at set intervals. The process of systematic management was followed by bug fixes, browser updates, and minor improvements due to user feedback. The platform’s modular architecture makes it possible to perform easy updates and aligns with the future integration of other hospital information systems.

## Results

The data included 50 patients in the time and motion assessment, 20 patients for detailed evaluation of service time at registration counters, and 200 patients who participated in a structured questionnaire to assess their satisfaction, knowledge, and willingness to accept digital registration.

### Demographic profile of respondents

Out of the 200 participants that filled the questionnaire survey, 54.5% were females and 45.5% were males, which indicates a quite balanced sex ratio. The respondents were both patients and family members accompanying them and visiting the Outpatient Department (OPD) of Kasturba Hospital from Monday to Saturday. Individuals in the Emergency Department, as well as those aged less than 18 years or 65 years and older, were excluded from the study. The patient age span was quite wide, from young adults to older persons, which featured the demographic of people who would probably use OPD services. Such a heterogeneous participant profile was a factor in creating a more accurate reflection of people’s opinions about the hospital’s registration process (Table 1).

**Table 1:** Demographics of respondents included in the study

**1a. Demographics of the end-users (** ***N*** ** = 200)**	
**Variables**	**Value**
Age in years (Mean ± SD)	50.53±$7.78$
**Gender** (%) Male Female	91 (45.5%) 109 (54.5%)
**Qualification** Accountant Teacher/Lecturer Bank manager Bank employee AC/Electrical Technician Secondary school General Duty worker Professional degree Not disclosed	6 (3%) 17 (8.5%) 4 (2%) 19 (9.5%) 8 (4%) 24 (12%) 13 (6.5%) 57 (28.5%) 52 (26%)
**1b. Demographics of the participants of total time spent at the registration counter (** ***n*** ** = 50)**	
Age in years (Mean ± SD)	51.1±$7.97$
**Gender** Male Female	32 (64%) 18 (36%)
**Occupation** Teacher/Lecturer Bank employee Security Hospital Technician Clerical staff Not disclosed	4 (8%) 5 (10%) 1 (2%) 3 (6%) 16 (32%) 21 (42%)
**1c. Demographics of the participants for completion of registration proceedings (** ***N*** ** = 20)**	
Age in years (Mean ± SD)	53.1±$7.03$
**Gender** (%) Male Female	12 (60%) 8 (40%)
**Occupation** Nurse School bus driver Teacher Doctor Hospital GD Staff Not disclosed	2 (10%) 1 (5%) 3 (15%) 1 (5%) 1 (5%) 12 (60%)

### Time and motion study

**Fig. 4:**
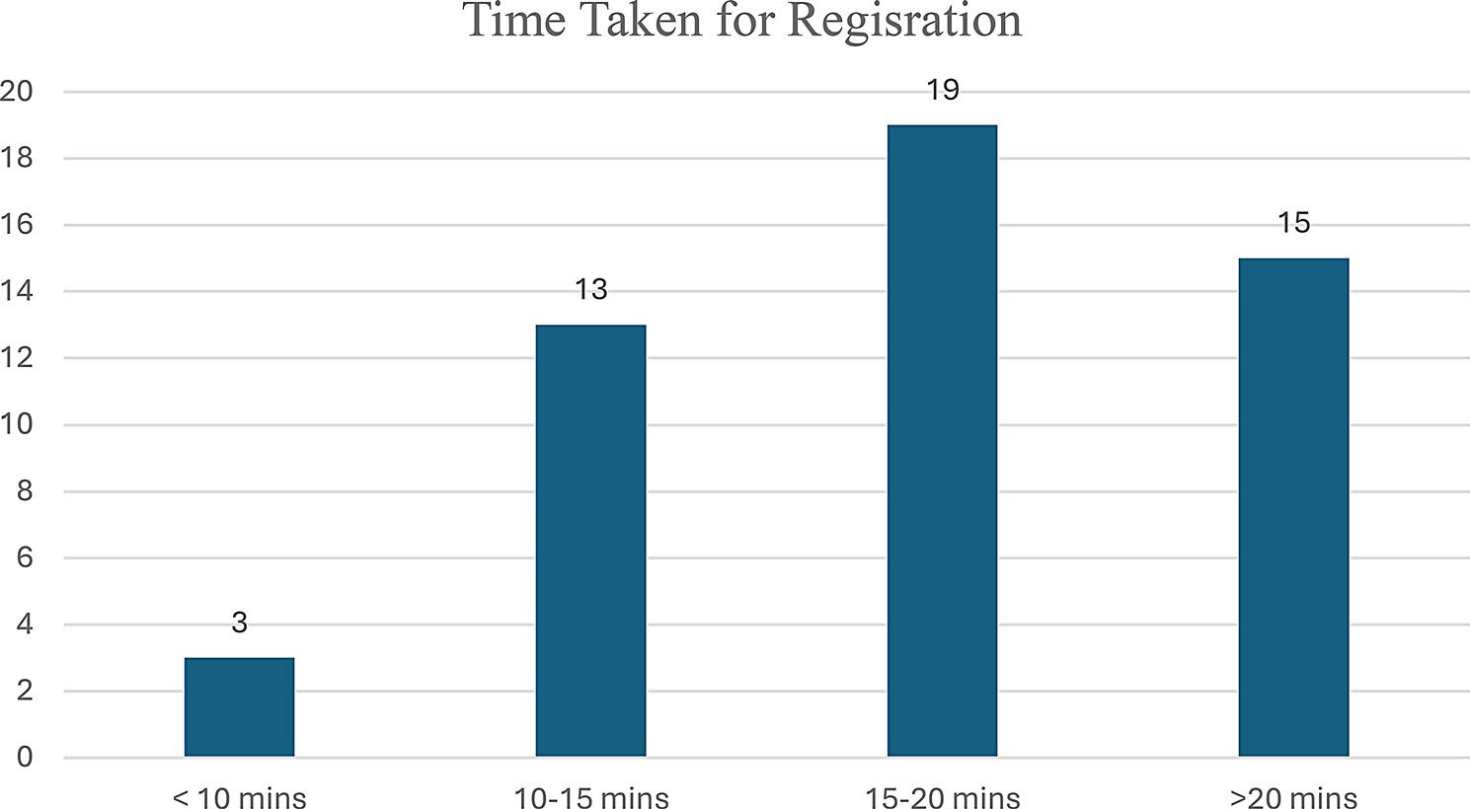
Frequency distribution of patient registration duration in time-motion study (*n* = 50)

The observational phase of the study documented the duration of the patients’ waiting times at the manual registration stage. Based on data from 50 patients, the average total waiting time from arriving at the registration desk to finishing the registration procedure was calculated to be 17 min and 25 s. This time comprises queue waiting and staff interaction (Fig. 4). In addition, an extra sample of 20 patients was analysed in order to determine the average service time taken by the registration counter staff, which was set at 6 min and 2 s. This service time corresponds to the staff’s task duration for detail checking, patient card issuing and directing patients to the right department. The differences in registration times reveal the operational inefficiencies of the paper-based system. Peak hour congestion together with reliance on manual data entry are the main reasons behind the prolonged waiting times, especially during mornings.

### Patient satisfaction with existing registration system

A structured questionnaire was distributed to 200 patients to evaluate their satisfaction with the outpatient registration workflow. The results supported that 84.5% of the patients knew about the registration process of the hospital. When the patients were asked for their opinions overall satisfaction, 74.5% told that they were satisfied with the paper-based system while a noTable 25.5% were not satisfied, giving the long waiting times and inefficiency of the processes as the reason. Among the factors affecting satisfaction, waiting time was the most influential. Only 13% of the patients could complete the registration in under five minutes, while 38.5% reported having to wait five to ten minutes. This suggests that there is a lot of variability in the registration process especially during peak times. The overall satisfaction of the patients with waiting times at varying service counters also reflected this delay. While 40.5% of patients expressed satisfaction, 26% remained neutral. In contrast, 19.5% reported dissatisfaction, and 9% were very dissatisfied (Table 2).

**Table 2:** Patient responses on existing and proposed outpatient registration system

Quantitative data presentation			
SN	Category	Values	Percentage
1	Are you aware of the registration process in Kasturba Hospital?	Yes No	84.5% 15.5%
2	Are you satisfied with the existing registration process?	Yes No	74.5% 25.5%
3	How long has it taken for the registration process?	1–5 min 5–10 min 11–15 min Above 15 min	22% 38.5% 26.5% 13%
4	How was the overall experience in the hospital in terms of waiting time at different counters since your arrival?	Very Satisfied Satisfied Neutral Dissatisfied Very Dissatisfied	5% 40.5% 26% 19.5% 9%
5	How was the experience in the queue at the registration counter?	Very Satisfied Satisfied Neutral Dissatisfied Very Dissatisfied	5.5% 40.5% 25.5% 24% 4.5%
6	How much would you rate the registration process?	Excellent Fair Good Poor	18.5% 31% 45.5% 5%
7	Are you aware of Online registration	Yes No	42% 58%
8	Do you think online registration will reduce the waiting time?	Yes No	83% 17%
9	Would you register online if the online registration website is developed?	Yes No	90.5% 9.5%

### Awareness and willingness to adopt online registration

Although digital health systems offer clear advantages, just 42% of the surveyed individuals declared having heard of an online registration system existing in the hospital. Such a low awareness indicates a vast communication and outreach gap. On the other hand, the data set revealed that patients were open to digital solutions: 90.5% of patients would be willing to take part in an online registration system if it was developed and implemented. In addition, 83% concurred that such a registration system would be a significant solution to the waiting time problem in the registration process itself. Therefore, the results pointed out that there was a clear acceptance of the digital-based healthcare services by the patient population provided that the system is user-friendly and well-promoted.

## Discussion

The Outpatient Department (OPD) is an important part of the hospital infrastructure and medical service providers, and it also creates the first impression of the healthcare service for patients. Besides, the OPD in tertiary care settings, especially in developing countries, is usually challenged with factors like patient overload, lack of adequate staff, poor infrastructure, and inefficient workflows among administration [17]. Increased waiting times, operational delays, and patient dissatisfaction are the consequences of these difficulties collaborating. Therefore, the main aim of our research was to look into the old-fashioned method of patient registration at Kasturba Hospital Udupi which involves the use of paper and to also examine the possibility and acceptance of a web-based online registration system as a digital alternative. The results of our survey indicated that 25.5% of the patients were not satisfied with the registration process mainly due to long waiting times. Meanwhile, 74.5% of patients showed their satisfaction, but the fact that 25% of patients were very dissatisfied cannot be ignored. This situation is similar to the negative impact that administrative delays have on the overall service perception in healthcare delivery. Sengupta et al. conducted studies in rural and semi-urban India where they found that patients often judge their entire hospital experience by the long waits at registration counters before they have even seen a doctor [15]. These situations result in overcrowding, inefficiencies, and at times even delays in critical consultations. A related study conducted by Binsar and Legowo investigated the SMS-based outpatient registration that was linked to hospital systems through the TOGAF ADM enterprise architecture framework. Their model was developed and implemented in a rural Indonesian hospital where patients, many of them without smartphones, were able to register remotely by texting and getting queue numbers and schedule confirmations. The main goal of the system was to cut down on the waiting time for manual registration, synchronize patient entries with the doctors’ availability, and link the process to BPJS (the health insurance system) through cloud validation. The authors illustrated that even in resource-poor environments, basic mobile technology could transform the processes of registering and largely satisfying the patients [18]. This evidence backs the current study’s focus on the context-specific, lightweight digital interventions for ameliorating outpatient flow in public hospitals.

In addition, the usability of the system and the motivation for its adoption are significantly linked to the way humans behave. Gamification was a tool that Binsar et al. used to create a framework aimed at healthcare staff in health information systems (HIS) activities [19]. Even though our study did not resort to gamification, the main idea, involving active stakeholder engagement, remained intact using patient feedback in the design of the system. Taking into consideration the user acceptance and usability, the socio-technical lens should be applied. Interoperability, trust, and interpretability are the core themes in the future of digital health architectures from a technology spectrum view. Binsar et al. in their review, proposed a CoT-XAI framework (Cognitive of Things with explainable AI) for assuring trust and clarity in medical IoT systems [20]. The current system does not include either AI or IoT; however, the long-term vision comprises factors such as appointment prediction, intelligent triage, and mobile health records integration. The implementation of these technologies will not only require adherence to ethical standards but also the establishment of trust via guidance from frameworks like CoT-XAI especially among patients who are digitally literate to various degrees. The literature keeps highlighting that the best practice in digital health is to design and implement technology around the needs and socio-cultural environment of the users.

The outcome of this investigation also confirms the incumbent technology adoption models. The patients’ affirmative reactions towards the internet-based system resonate with the essential concepts of the Technology Acceptance Model [21], mainly perceived utility and perceived ease of use. Dissatisfaction with long wait times supports the need for a digital alternative, while the friendliness of the interface supports the ease of use. In the context of the Unified Theory of Acceptance and Use of Technology [22], the system was taken in by the staff assistance, integration with existing practices, and the requirement for minimal infrastructure. Digital health adoption models also take organizational readiness, workflow integration, and user trust into account as key factors contributing to the success of the adoption process. The system’s lightweight design, browser plugin for seamless integration, and positive patient feedback are in line with the theoretical determinants of digital health adoption.

## Conclusion

To conclude, outpatient registration system of a tertiary care hospital which is still paper based, the observations that have been done reveal an average registration time of 17 min. Roughly 25% of the participants were dissatisfied with the time they spent waiting, so direct inefficiencies in service delivery were exposed. Moreover, the openness of patients to the new technology was very high, 90.5% were ready to register online and 83% believed such a system would help decrease waiting times. The groundwork for these insights was laid, and a mobile-friendly online registration system was designed and partially implemented the waterfall development approach was used. The hospital’s already existing health information platform was technically integrated with the system and automated data entry through a backend browser extension was included. While the benefits of such an improvement like reduced congestion and more efficient service may be seen, the outcomes of these were not part of the evaluation in the current study. In other words, the conclusions focus on perceived system delays, the positive attitude of patients toward digitization, and the successful development of a working prototype. The next step for researchers will be to do full-scale deployment and post-implementation assessment to find out the actual impact and sustainability.

## Strength and limitations

There are some notable strengths in this study. Set up in a tertiary care hospital, it fits well in a real-world clinical environment, maintaining the study relevant amid current outpatient workflows. The use of a prospective observational study, combining a time-and-motion study with a structured patient satisfaction survey, allowed us to capture both objective operational metrics and subjective patient perspectives. The mobile online patient registration system was designed out directly from patient feedback, thus representing a much more patient-centered and context-specific solution. Technically implemented using Django framework and MySQL database and a browser plugin to integrate into the hospital system, this study provided a proof of concept to show how such a system could be deployed in similar healthcare settings. The large sample size of 200 surveyed participants gave a wider perspective from patients which, in turn, made the findings more reliable. Yet there are limitations to this study. It is a single-centre study and is carried out in a hospital in coastal Karnataka, so perhaps the results cannot be generalized to other geographic and institutional contexts. The study did not account for patients’ digital literacy or access to smartphones, which are crucial determinants of actual adoption despite expressed willingness. The study used convenience sampling, which could introduce selection bias. Additionally, there was an absence of post-implementation evaluation, so the long-term impact and actual adoption of the system could not be assessed. Lastly, the absence of a control or comparison group using a functioning digital system restricts the ability to benchmark improvements.

## Supplementary Information

Below is the link to the electronic supplementary material.

Supplementary Material 1

## Data Availability

The datasets used and/or analysed during the current study are available from the corresponding author upon reasonable request.
