# Supplementary material for: Design and implementation of a web-based patient registration system in a single-centered tertiary care hospital of coastal Karnataka
Source: BMC Health Serv Res. 2026 Mar 10;26:533. doi: 10.1186/s12913-026-14299-3 (PMC13085711; doi:10.1186/s12913-026-14299-3)
Supplement: Supplementary file 1 — Supplementary Material 1 [file 12913_2026_14299_MOESM1_ESM.pdf]

Development of an Online Registration Website in a Tertiary Care Hospital of Coastal Karnataka

Respondents Detail:

Name: ________________________ Sex: M/F Age:

Occupation: ________________________________

Aim: To develop mobile-based Out-Patient registration website for patients.

Objectives:

- To study the patient waiting time in the registration area
- To assess the patient's willingness to adapt to online registration system
- To develop a mobile-based outpatient registration website for patients
- To assess the effectiveness of the website

Instructions: Respondents are requested to go through the questionnaire and provide their opinion by marking (√) against the specific option.

1. Are you aware of the registration process in Kasturba Hospital?
   ☐ Yes ☐ No
2. Are you satisfied with the existing registration process?
   ☐ Yes ☐ No
3. How long had it taken for the registration process?
   ☐ Less than 5 min
   ☐ 5–10 min
   ☐ 11-15 min
   ☐ Above 15 min
4. How was your overall experience in the hospital in terms of waiting time at different counters since your arrival?
   ☐ Very dissatisfied
   ☐ Dissatisfied
   ☐ Neutral
   ☐ Satisfied
   ☐ Very satisfied
5. How was your experience in the queue at the registration counter?
   ☐ Very dissatisfied
   ☐ Dissatisfied
   ☐ Neutral
   ☐ Satisfied
   ☐ Very satisfied

6. How much would you rate the registration process?
   ☐ Excellent
   ☐ Good
   ☐ Fair
   ☐ Poor
   ☐ Very Poor
7. Are you aware of online registration?
   ☐ Yes ☐ No
8. Do you think online registration will reduce the waiting time?
   ☐ Yes ☐ No
9. Would you register online if the online registration website is developed?
   ☐ Yes ☐ No

****
